# The Implementation of a Primary Care-Based Integrated Mobile Health Intervention for Stroke Management in Rural China: Mixed-Methods Process Evaluation

**DOI:** 10.3389/fpubh.2021.774907

**Published:** 2021-11-17

**Authors:** Enying Gong, Lixin Sun, Qian Long, Hanzhang Xu, Wanbing Gu, Janet Prvu Bettger, Jingru Tan, Jixiang Ma, Tazeen Hasan Jafar, Brian Oldenburg, Lijing L. Yan

**Affiliations:** ^1^School of Population Medicine and Public Health, China Academy of Medical Science & Peking Union Medical College, Beijing, China; ^2^Global Health Research Center, Duke Kunshan University, Suzhou, China; ^3^School of Population and Global Health, The University of Melbourne, Melbourne, VIC, Australia; ^4^Duke Global Health Institute, Duke University, Durham, NC, United States; ^5^Department of Orthopedic Surgery, Duke University, Durham, NC, United States; ^6^Shandong Center for Disease Control and Prevention, Shandong, China; ^7^Program in Health Services & Systems Research, Duke-NUS Medical School, Singapore, Singapore; ^8^Non-communicable Disease Unit, Baker Institute, Melbourne, VIC, Australia; ^9^School of Health Sciences, Wuhan University, Wuhan, China; ^10^Peking University School of Global Health and Development, Peking University, Beijing, China

**Keywords:** stroke, implementation evaluation, mobile health, rural China, RE-AIM (reach, effectiveness, adoption, implementation and maintenance)

## Abstract

**Background:** There is a lack of evidence concerning the effective implementation of strategies for stroke prevention and management, particularly in resource-limited settings. A primary-care-based integrated mobile health intervention (SINEMA intervention) has been implemented and evaluated *via* a 1-year-long cluster-randomized controlled trial. This study reports the findings from the trial implementation and process evaluation that investigate the implementation of the intervention and inform factors that may influence the wider implementation of the intervention in the future.

**Methods:** We developed an evaluation framework by employing both the RE-AIM framework and the MRC process evaluation framework to describe the implementation indicators, related enablers and barriers, and illustrate some potential impact pathways that may influence the effectiveness of the intervention in the trial. Quantitative data were collected from surveys and extracted from digital health monitoring systems. In addition, we conducted quarterly in-depth interviews with stakeholders in order to understand barriers and enablers of program implementation and effectiveness. Quantitative data analysis and thematic qualitative data analysis were applied, and the findings were synthesized based on the evaluation framework.

**Results:** The SINEMA intervention was successfully implemented in 25 rural villages, reached 637 patients with stroke in rural Northern China during the 12 months of the trial. Almost 90% of the participants received all follow-up visits per protocol, and about half of the participants received daily voice messages. The majority of the intervention components were adopted by village doctors with some adaptation made. The interaction between human-delivered and technology-enabled components reinforced the program implementation and effectiveness. However, characteristics of the participants, doctor-patient relationships, and the healthcare system context attributed to the variation of program implementation and effectiveness.

**Conclusion:** A comprehensive evaluation of program implementation demonstrates that the SINEMA program was well implemented in rural China. Findings from this research provide additional information for program adaptation, which shed light on the future program scale-up. The study also demonstrates the feasibility of combining RE-AIM and MRC process evaluation frameworks in process and implementation evaluation in trials.

**Clinical Trial Registration:** www.ClinicalTrials.gov, identifier: NCT03185858.

## Introduction

Stroke is the second leading cause of mortality and disability worldwide. An estimation from the Global Burden of Disease study found that 77% of stroke survivors were from low- and middle-income countries (LMICs), where individuals with lower socioeconomic status suffer more, and effective strategy in secondary prevention is far lacking (1, 2). In China, the burden of stroke is substantial, with 11.1 million stroke survivors national wide (3, 4) and disproportionally higher in Northern China and rural regions (5). The recurrence rate was as high as 11.2% for all stroke survivors and was 5.7% within 1 year and 22.5% within 5 years among the low-income rural population (5, 6). The limited capacity of primary healthcare system and the overburden of secondary and tertiary hospitals attributed to the fragmented care for stroke prevention in rural China. Thus, effective strategies to improve stroke management are in great need.

Evidence on effective secondary prevention exists, but challenges lie in translating these strategies into routine practice, especially in resource-constrained settings. A primary care-based integrated mobile health intervention (SINEMA intervention) has been designed and implemented in rural China (7). This intervention applied tailored intervention strategies tested in previous programs, such as task shifting, task sharing, and digital health technologies (8–11), and targeted primary healthcare providers and community-dwelling patients who suffered stroke to address the barriers in stroke management. The effectiveness of the SINEMA intervention has been demonstrated, with improvements in blood pressure control, medication adherence and quality of life, and a reduction in disability, stroke recurrence, and deaths were also observed at 12 months among stroke survivors, allocated to the intervention arm compared with the participants who received usual care (12).

Further investigation of the implementation of the SINEMA intervention is very important in order to uncover the implementation outcomes and understand the extent to which effectiveness was affected by other factors (13, 14). Such findings will also help inform the future optimization of implementation of the SINEMA intervention in other settings. Many different frameworks have been proposed for guiding the implementation evaluation (14–18). For example, the RE-AIM evaluation framework proposes five key dimensions—with these being, reach, effectiveness, adoption, implementation, and maintenance—to inform the future implementation, generalizability, and scalability of effective programs (17). The MRC process evaluation framework is another commonly used framework for complex intervention that emphasizes the implementation, mechanisms of impact, contextual factors, and the relationships between these dimensions (14). While many different community-based interventions have adopted a single framework to illustrate certain aspects of program implementation (15), few studies have undertaken a comprehensive evaluation by utilizing quantitative and qualitative data to describe the implementation outcomes and explain how the program was implemented.

This current study examines the implementation of the SINEMA intervention to provide further information for researchers, practitioners, and policymakers. We developed an implementation and process an evaluation framework that combined both the RE-AIM and MRC process evaluation frameworks and utilized both quantitative and qualitative data. This paper reports the findings on implementation outcomes, relates enablers and barriers, and illustrates some potential impact pathways that may influence the effectiveness of the SINEMA intervention and its wider implementation.

## Methods

### SINEMA Trial, Study Setting, and Intervention Components

The SINEMA trial was a Hybrid II effectiveness-implementation trial (19). The effectiveness of the SINEMA program was investigated by a cluster randomized controlled trial conducted among 50 villages of rural Hebei Province, Northern China (7). A total of 1,299 rural stroke survivors (an average of 25.5 participants per village) were recruited in the trial. Twenty-five villages, including 637 patients, were randomly allocated in the intervention arm and implemented the SINEMA program over 12 months (12).

The study was conducted in a resource-limited rural county with doubled stroke burden and less than half of the annual disposable income *per capita* than the national average (20–22). In rural China, the general practice and preventive care are mainly delivered by primary healthcare providers, including village doctors who have received minimum basic medical and pharmaceutical training and can prescribe medications (23). The acute-stage stroke treatments are mainly delivered at county hospitals, while rehabilitative care and follow-up visits are largely unavailable. The outpatient services are paid out of pocket, although the zero-Markup drug policy allows low price of medications in the village clinics and the NCD insurance package reduced the catastrophic health expenditure by reimbursing some outpatient services if patients received care from county hospitals (24).

**Figure 1:**
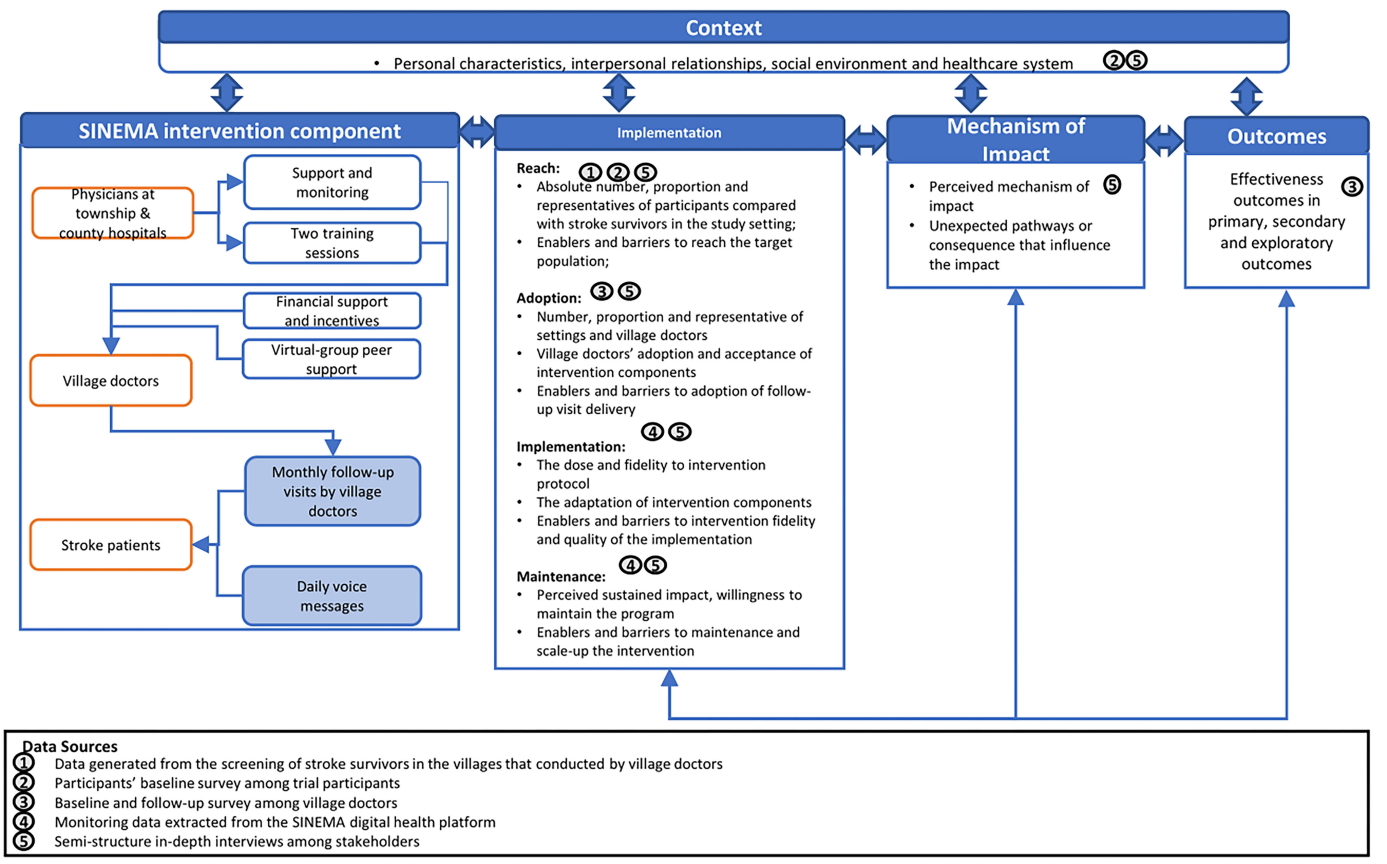
Evaluation framework.

Built on such context, the SINEMA intervention was designed, and a detailed description of the intervention and trial design has already been published elsewhere (7). In brief, the SINEMA intervention included both provider-facing and patient-facing components (Figure 1, left panel). As both receptors and providers of the intervention, village doctors received training, performance-based financial support, and virtual-group peer support. They delivered monthly face-to-face follow-up visits to participants with support from an android-based mobile application (*SINEMA App*). The participants received monthly follow-up visits and daily voice messages dispatched automatically at no cost if they had a phone available. A digital health system, consisting of the SINEMA App and voice messages dispatching system, was developed to support the program delivery (25, 26). Besides, five physicians from township hospitals and one county manager also facilitated the program implementation by providing support and performing quality control.

### Evaluation Framework and key Measurement

The implementation and process evaluation was performed based on an evaluation framework that derived from both the RE-AIM framework (17) and the MRC process evaluation framework (14), as described in Figure 1. The RE-AIM framework was used to inform the measurement of implementation outcomes, covering program reach, adoption, implementation, and maintenance. Reach was assessed by the absolute proportion and the representativeness of individuals involved in the trial among those identified with stroke history during the village-wide screening. Adoption was measured from the provider perspective by considering the acceptance and uptake of the intervention among village doctors. Implementation was evaluated both quantitatively and qualitatively, including the intensity and the quality of services delivered and the adaptation made by providers. Maintenance was defined as perceived willingness of providers and participants to maintain or scale up the SINEMA program post-trial. We also identified facilitators and barriers that may influence each RE-AIM dimensions. The MRC process evaluation framework was used to investigate the interactive relationship between context, intervention components, and implementation indicators. The MRC framework also enabled us to assess some of the unexpected pathways or consequences that have not been previously considered.

### Data Collection

To obtain a comprehensive overview of program implementation, we collected data in multiple phases from various stakeholders. Multiple data collection approaches were adopted, including self-administered surveys among village doctors, face-to-face interview-based surveys among participants, monitoring data from the digital health system, and in-depth interviews among stakeholders. Table 1 summarizes the data source and data collection approaches.

**Table 1:** Data sources and data collection approach.

**Stage of the program implementation**	**Timepoint of data collection**	**Type of data collection**	**Information collected**	**Data collection approach**	**Type of data**
Pre-implementation	Screening (before 0 months)	Screening data among potential participants	Basic information (socio-demographic, stroke history) of stroke survivors (60 villages, *n* = 2333)	Village doctors filled in the form based on existing health records or interviews with potential participants.	Quan
	Baseline (0 months)	Survey among recruited participants	Basic information and major outcome indicators of recruited participants' (50 villages, *n* = 1,299)	A questionnaire administered by trained assessors via face-to-face interviews.	Quan
	Baseline (0 months)	Self-administered surveys among village doctors	Basic information, attitude and practice of stroke prevention among village doctors (*n* = 50)	Village doctors self-administered the online survey.	Quan
Implementation	Throughout program implementation (0–12 months)	Monitoring data from digital health system	The number of follow-up visits delivered, and voice messages received.	Extracted from digital health platforms.	Quan
	Quarterly (3, 6, 9 months)	Semi-structured in-depth interviews among stakeholders	Implementation situation and relevant factors among participants, village doctors, township physicians.	Project-related researchers conducted semi-structure in-depth interviews.	Qual
Post-implementation	Quarterly (12 months)	Semi-structured in-depth interviews among stakeholders	Implementation situation and relevant factors among participants, village doctors, township physicians.	Researchers who have not involved in the program design and implementation conducted semi-structured in-depth interviews.	Qual
	Follow-up survey (12 months)	Self-administered surveys among village doctors	Attitude and practice of stroke management among 50 village doctors, acceptance and adoption of intervention components among 25 village doctors in intervention arm.	Village doctors self-administered the online survey.	Quan

#### Self-Administered Surveys Among Village Doctors

Fifty village doctors participated in a self-administered survey dispatched *via* an online survey platform (Qualtrics, Provo, UT) at both baseline and 12 months. The survey asked about their sociodemographic information and their knowledge, attitude, and practice in the secondary prevention of stroke. Questions about the acceptance and preference in intervention components were also asked at the 12 months among village doctors allocated in the intervention arm.

#### Screening Data About Potential Participants

Before recruitment of participants, village doctors conducted a screening of residents in the villages to identify people who had been diagnosed with stroke. Village doctors reviewed health records of existing residents or conducted door-to-door screening and provided a list of potential participants with detailed information on age, gender, stroke history, basic communication abilities, and disabilities. This information guided the invitation of the potential participants and provided the information to understand the reach of the program.

#### Surveys Among Recruited Participants

Among participants, trained assessors performed a face-to-face baseline survey. These data covered a broad range of information, including their sociodemographic characteristics and a series of indicators on health history, health behaviors, and health conditions.

#### Monitoring Data From Digital Health System

The digital health system, which consisted of the SINEMA mobile application and voice-message dispatching system, also tracked and monitored the delivery of follow-up visits and voice messages. The data on follow-up were extracted from the SINEMA server, which tracked the number of follow-up visits delivered by each village doctor and for each patient. A third-party message-dispatching system captured the number of voice messages distributed and recorded the number of voice messages answered by the participants on a given day.

#### Semi-structured In-depth Interviews Among Stakeholders

Semi-structured in-depth interviews were conducted for four waves at the 3rd, 6th, 9th, and 12th months from the initial of the intervention with slightly different purposes and adjusted interview guides. Supplementary Table 1 reported the number of interviews conducted in each wave and by type of stakeholders. The first two waves focused on how the SINEMA intervention was adopted and implemented and the potential enablers and barriers that may influence the implementation. The third and fourth waves of in-depth interviews focused on the impact of intervention components and the key enablers and barriers that may influence the implementation, effectiveness, and maintenance. The first three waves of interviews were conducted by the research team, while the last wave was performed by an independent investigator who was not involved in any stage of the intervention design and implementation to ensure the objectivity of the evaluation. Stakeholders involved in the intervention implementation (including patients who suffered stroke, village doctors, physicians at township hospitals, and a county coordinator) were invited to participate in the interviews in all four waves. The village doctors who allocated in the control arm were also invited at the fourth wave.

We used purposive sampling to ensure the diversity of the participants and the coverage across villages and townships. At each wave of data collection, the research team identified the villages to ensure that at least one village from each township was selected. All intervention villages were covered across the four waves of data collection. Within each village, the research team interviewed the village doctor and selected one or two participants based on their availability, willingness, ability to communicate, and demographic characteristics to ensure representatives of the participants. Interviews were conducted either in the village clinics or at the homes of the participants. The physicians at township hospitals and the county coordinator who involved in the study were also interviewed at their working places. All interviews lasted between 20 and 40 min and were audio-recorded with verbatim transcripts for data analysis.

### Data Analysis

The data analysis of quantitative and qualitative data was performed independently, and then the findings were embedded within the designed evaluation framework (27). This approach gathered the quantitative and qualitative data by not only demonstrating the key dimensions of program implementation but also exploring its variations, facilitators, and barriers. For quantitative data, descriptive analysis was performed by using STATA 15.0 software. *T*-test and chi-square test were used for comparison between groups of participation status.

For qualitative data, the thematic analysis approach was applied with the following steps: First, we familiarized ourselves with the data by reading all transcripts. Due to the large numbers of interviews administered during the four waves (*n* = 98), we went through all the transcripts and classified the quality of the transcripts based on the quality of the interview, the amount of information contained in the conversation, the types of stakeholders, and the wave of data collection. Forty-three (43.8%) transcripts were classified into the high-quality group and received full analysis with line-by-line coding from two researchers, while others received rapid coding from one researcher (Supplementary Table 1 reports the number of transcripts involved in the full analysis). Second, we developed the coding structure. Researchers (EG, LS) coded at least one transcript from each type of stakeholders from all four waves. Data were coded on a line-by-line basis, and data were initially organized according to the topic of questions from the interview guide. We then inductively derived codes with a more elaborate hierarchical coding scheme by considering different intervention components and the dimensions of the evaluation framework. Researchers discussed the coding structure, and issues were resolved by consensus. Third, transcripts classified as high-quality transcripts were coded by two researchers independently, with at least half transcripts were double coded to ensure the objectivity and transparency of the process. Any discrepant interpretations were discussed between the researchers and across a broader research team. The researchers also scanned the remaining transcripts that were classified as low-quality transcripts to avoid missing information. Fourth, themes were developed to map each dimension of the framework by reading the coded data and the original transcripts to ensure that the themes were authentic and rooted in the data. All the quotes involved in the manuscript were translated from Chinese to English.

## Results

Utilizing the evaluation framework described in Figure 1, we summarized the context, implementation outcomes, and impact pathways of the SINEMA intervention. Characteristics of stakeholders involved in the in-depth interviews are summarized in Tables 2, 3.

**Table 2:** The characteristics of the participants who were involved in the screening, recruited in the trial, and participated in the in-depth interviews.

**Characteristics of participants**	**Stroke survivors screened ^a^** **(*n* = 2,081)**	**Stroke survivors participated in the trial** **(*n* = 1,299)**	**Stroke survivors involved in the interviews** **(*n* = 51)**
Sex, % female	909 (43.7%)	553 (42.6%)	23 (45.1%)
Mean age at baseline (SD)	67.1 (9.2)	65.7 (8.2)	65.6 (7.7)
**Stroke type ^b^**			
Ischemic	1,731 (83.2%)	1,119 (86.1%)	42 (82.4%)
Hemorrhage	331 (15.9%)	176 (13.6%)	9 (17.6%)
Not specified	19 (0.9%)	4 (0.3%)	0 (0%)
**Self-report medicine taking during screening or baseline survey**			
Antiplatelet	1,357 (65.2%)	852 (65.6%)	19 (37.3%)
Satin	699 (33.6%)	340 (26.2%)	11 (21.6%)
Anti-hypertensive medicines	1,675 (80.5%)	1,030 (79.3%)	42 (82.4%)
Had experienced stroke recurrence	603 (29.0%)	378 (29.1%)	10 (19.6%)
Visited to village clinics in the past month	1,600 (76.9%)	795 (61.2%)	40 (78.4%)
Difficult to get out of bed ^c^	160 (7.7%)	27 (2.1%)	0 (0%)
Having basic communication ability	1,919 (92.2%)	1,259 (96.9%)	50 (98.0%)
**Duration since the first stroke event**			
<3 years	549 (26.4%)	357 (27.5%)	10 (19.6%)
3–5 years	443 (21.3%)	329 (25.3%)	18 (35.3%)
6–9 years	479 (23.0%)	257 (19.8%)	7 (13.7%)
≥ 10 years	610 (29.3%)	356 (27.4%)	16 (31.4%)

a *Only stroke survivors who were from the 50 eligible villages were accounted. Stroke survivors from 10 villages that did not meet the cluster eligible criteria were excluded from the analysis*.

b *For the participants who had multiple stroke experiences, the type of stroke accounted for the latest stroke event*.

c *The participants who had limited walkability but able to visit to the village clinics with support of family caregivers were included in the trial, otherwise were excluded*.

**Table 3:** Characteristics of village doctors who were involved in trial, implemented the SINEMA intervention, and participated in the in-depth interviews and their perceptions of stroke care and SINEMA intervention components.

**Characteristics and perceptions of village doctors**	**Involved in the trial** **(*n* = 50)**	**Allocated in intervention arm** **(*n* = 25)**	**Participated in-depth interviews** **(*n* = 27)**
**Age, mean (SD), years**	46.0 (6.4)	46.1 (7.3)	46.0 (7.5)
**Sex, % female**	8 (16.0%)	3 (12.0%)	3 (11.1%)
**Education**, ***n*** **(%)**			
High school or equivalent	29 (58.0%)	15 (60.0%)	16 (59.3%)
Junior college	18 (36.0%)	8 (32.0%)	9 (33.3%)
College	3 (6.0%)	2 (8.0%)	2 (7.4%)
**Years as village doctor, mean (SD)**	24.3 (7.4)	24.2 (8.6)	24.2 (8.8)
**Self-evaluation on existing workload at baseline**			
Very high	12 (24.0%)	6 (24.0%)	6 (22.2%)
High	26 (52.0%)	12 (48.0%)	13 (48.1%)
Acceptable	12 (24.0%)	7 (28.0%)	8 (29.6%)
**Agreement on the top three most essential intervention components**			
Face-to-face training	NA	20 (80.0%)	NA
SINEMA App (training module)	NA	13 (52.0%)	NA
SINEMA App (follow-up module)	NA	14 (56.0%)	NA
SINEMA App (reminder module)	NA	5 (20.0%)	NA
SINEMA App (performance statistics module)	NA	3 (12.0%)	NA
Financial compensations and incentives	NA	4 (16.0%)	NA
Reminders and feedbacks from township physicians	NA	7 (28.0%)	NA
**Agreement on the following statements related to the perceptions and attitude on stroke care at 12 months of the intervention implementation**			
I am aware of the health conditions of stroke patients in my village.	33 (67.4%)	18 (75.0%)	19 (70.4%)
I am confidence in prescribing the most appropriate medicines for stroke patients.	41 (83.7%)	21 (87.5%)	21 (77.8%)
I am confident in providing support and guidance to stroke patients.	42 (85.7%)	24 (100.0%)	25 (92.6%)
My patients trusted me.	39 (79.6%)	22 (91.7%)	23 (85.2%)
All my stroke patients could adhere to my suggestions and prescriptions.	24 (49.0%)	15 (62.5%)	15 (55.6%)
**Agreement on the following statements related to the impact of the intervention at the 12 months of the intervention implementation**			
The frequency of getting blood pressure monitoring had improved among my patients.	NA	22 (91.7%)	NA
The program led to a clear improvement in blood pressure control among my patients.	NA	23 (95.8%)	NA
The program led to a clear improvement in medication adherence among my patients.	NA	23 (95.8%)	NA
The program made more patients in my villages proactively do physical activities.	NA	24 (100.0%)	NA
My patients rely on me more after the project.	NA	21 (87.5%)	NA
The project improved my authority in the village.	NA	21 (87.5%)	NA

*NA. not applicable, as the questions were only for village doctors in the intervention arm*.

### The Context for Implementing SINEMA

#### Characteristics of Villages and Healthcare System in the Region

The SINEMA intervention was implemented in Nanhe County, rural Hebei, China. The 50 villages in the rural regions scattered around urban areas of the county, where the county hospitals located with an average distance of 14.5 km (Supplementary Table 2). During the interview, majority of the participants identified village clinics as their first contact point of the healthcare system to address their day-to-day health needs; some participants also mentioned that they sought care from other healthcare facilities, including private clinics and pharmacies within the village or nearby villages, township hospitals, county hospitals, and hospitals in nearby cities. Several factors may determine different choices of healthcare facilities, such as healthcare needs of the participants, trust and relationship with doctors, insurance coverage, and the quality of available services.

*“I visited village clinics quite often. If I am available, I will come here to measure my blood pressure. Not every day, but one time per 3 to 5 days.” –Participant, 3-month interview*

*“I got my medicines from the No. 2 county hospital because I could get reimbursement from the hospital. I need to pay out of pocket if I get medicine from the village clinic.”*

*- Participant, 6-month interview*

#### Interpersonal Relationships and Support for Care

According to the baseline survey, 410 (64.3%) participants had a family caregiver, mainly their spouses, daughters or daughters-in-law, as most of their sons or sons-in-law were working outside of villages. Family caregivers played the most critical role in daily life and treatment adherence of the participants. Although the participants mentioned that they know other village residents pretty well, people who had insufficient support from the family members could not get extra help from other neighbors or friends as stigma related to stroke existed. Some participants mentioned that they were unwilling to depend on other people or discuss their health conditions with other neighbors in the villages.

*“I don't know other people who also had this disease (stroke), but there should be some. I don't like to bother others. I could do most of the things by myself. I don't like to talk too much with others as I don't want to become a topic of their gossips.”– Participant, 6-month interview*

#### Personal Characteristics

At the personal level, the study participants were a vulnerable population group with low socioeconomic status. Among the participants who received the SINEMA intervention, 264 (41.1%) had received no formal schooling at all, 276 (43.3%) had more than two other chronic diseases, and 179 (28.1%) were experiencing moderate to severe disabilities (Table 2). About 7.3% participants experienced depression at the baseline, and many of them experienced various levels of cognitive impairment or other issues related to stroke or aging, which brought further obstacles to understanding and accepting the intervention, building their confidence, and improving self-efficacy and self-management behaviors.

*“My health condition is getting poorly, and I don't think I could be fully recovered as I am getting old anyhow.”—Participant, 3-month interview*

*“Some stroke patients had impaired brain function and poor memory. They thought there was no big difference whether they take medicine or not.”—Village doctor, 6-month interview*

### Implementation Outcomes

This section presents the results on implementation outcomes regarding program reach, adoption, implementation, and maintenance. The enablers and barriers that influence each domain of the implementation outcomes are summarized in Table 4 and described briefly below.

**Table 4:** Enablers and barriers on implementation indicators.

**Indicators**	**Enablers or Barriers**	**Some selected quotes**
Reach	***Enablers:*** • Knowledgeable community healthcare workers (village doctors) • Existing health records and door-to-door screening	• *“I had screened 35 potential participants, but there were a few patients who I failed to contact (during the screening and recruitment stage). A few of them were not at home when I tried to contact them, after several try, I gave them up. There is another patient who was hospitalized; thus, it ended up with 23 participants (after the eligibility screening).”—Vilage doctor 6, 9- month*
	***Barriers:*** • Participants' migration for working and family purposes	
Adoption of the SINEMA program among providers	***Enablers:*** • Knowledge, capabilities and confidence gained from training and previous experience • Perceived benefits on residents and themselves • Perceived credibility from the top-down approach	• *“The training sessions were helpful. I learned some new knowledge, which is helpful in my work. Especially the knowledge on the effect and side-effect of these essential medicines. Following what the chief physician told in training, I started to adjust the medicines for patients during the follow-up visits.”—Village doctor 2, 3-month* • *“I was in charge of the blood pressure management service (as part of the Basic Public Health Services). Even if there is no such program focus on stroke patients, I need to manage more than a hundred of patients with hypertension. Delivering follow-up visits (quarterly) is my job. Thus, for some of our participants, this intervention is an add-on service to the Basic Public Health Services.”—Village doctor 10, 12-months* • *“The financial compensation did not attract me at all. I participated because the township physicians invited me and said this program is good for our residents and I could also learn a lot from the program.”—Village doctor 8, 9-month*
	***Barriers:*** • Poor technology literacy • Lacking needs and motivations in specific tasks	• *“At the beginning, I wasn't too familiar with the app and the procedure, but I grasp the skills and could deliver follow-up visits smoothly after several rounds (of monthly visits).”—Village doctor 1, 6-month* • *“It (APP) reminded the date of follow-up visits for each patient, but I started follow-up visits before the system reminds me. It could be useful, but I don't need it.”— Village doctor 5, 12-month*
Implementation and fidelity of follow-up visits	***Enablers:*** • Patients' needs and willingness in improving health and Providers' responsibilities and efforts • Trusted doctor-patient relationships • SINEMA App standardized procedures • Support and quality control from township physicians	• *“People won't reject help on improving their health. So there is no much difficulties for me to implement the follow-up visits.”—Village doctor5, 12- month* • *“I manage all stroke patients in this village. They seldomly visit other providers. I know their health condition quite well and patients relies on me, and they get used to this relationship… This relationship was not established in a day; it has been several years.”—Village doctor 3, 12-month* • *“Before the program, I delivered services to patients, but there is no standard. I am now delivering follow-up visits by following the app. It is simple and comprehensive. The focus is not on the diagnosis, but on follow-up visits to communicate about their conditions. Having this procedure is helpful.”—Village doctor 1, 12-month* • *“For some village doctors, I need to remind them multiple times (to complete the follow-up visits). I checked their follow-up records to see whether there is any patient with extremely high blood pressure that needs more attention. I also talk to village doctors, if I found some unreasonable records.” —Township physician, 3-months*
	***Barriers:*** • Pre-existing heavy workload and competing programs • Participants' low awareness, adherence or cooperation • Technical difficulties (unstable internet access)	• *“The workload was increased, but I conduct follow-up visits when I was not too busy.”—Village doctor 3, 12-month* • *“For some patients, they don't give enough attention to it. You call them several times, but they do not come. For some people who are elderly or have low awareness, they don't care it too much. You have to visit them and let them know the importance.”—Village doctor 5, 3-month* • *“Sometimes, the internet was not stable, and the data cannot be uploaded. Thus, it is better to take the information down and then upload later.”—Village doctor 5, 6-month*
Implementation and fidelity of voice-message components	***Enablers:*** • Perceived benefits • Free at no cost • Simplicity in content and dispatch way • Nudges and suggestions from village doctors	• *“I received it (voice message) every day. I seldom had a phone call, but I receive your message every day. I learn things from listening to it, and there is no cost.”—Participant 14, 12-month* • *“I can receive it every day. It was useful for me. I put my phone near my bed. It reminds me of taking medicines and do exercise. It (voice messages) told me many knowledges and I followed it.”—Participant 3, 6-month* • *“During each follow-up visits, I will remind them to continue taking medicines and pick up the voice messages if they can.”—Village doctor 2, 12-month*
	***Barriers:*** • Phone ownership and use pattern • Lacking individualized contents to meet diverse needs • Hearing problems	• *“I have received voice messages, but not every day. Sometimes, I went out but didn't bring my phone with me; then I could not receive the messages.”—Participant 1, 3-month* • *“I don't use the phone quite often. I don't know how to use it. My phone shared with my family members. When it put here, I could listen to it; otherwise, I cannot. I have received some…. There was once when I clicked the button, but I cannot hear it.”- Participant 5, 3-month* • *“Some people they seldom pick up phone calls. Especially the elderly, they have hearing issues, and they cannot pick up the call.”—Township physician 1, 6-month*
Maintenance	***Enablers:*** • Perceived cost-benefits on patients and providers • Supportive environment Integration with other services	• *“I don't foresee too much challenge (in continuing the service). It is a basic service at no cost for patients. They will accept it… It (the SINEMA program) also didn't bring too much burden on me. At least, it helped the patient-doctor relationship. I also improved my skills by communicating more frequently with many patients.”—Village doctor 1, 12-month* • *“It also depends on the village doctors. Some village doctors who are more responsive to the patients in the village will continue doing it even without the program and financial support.” —County manager, 12-month* • *“This program should be integrated with other activities in both urban and rural settings. Such as the program should be integrated with the basic public health services so that the program could get more policy support.” —Township physician, 12-month*
	***Barriers:*** • Workloads and competing programs • Lacking mechanism to share the implementation cost	• *“If we included it (the SINEMA program) as routine services, the workload could be heavy. There are patients with stroke, coronary heart diseases, and mental disorders. If all counted, the workload will be heavy to maintain the current frequency of follow-up visits.”—Village doctor 4, 12-month* • *“It is quite important to identify how the cost could be sustainably covered. Maybe the program could be integrated with public health programs or other existing programs. It is hard to allocate funding if there is no support from the county or above authorities.”—Township physician, 12-month*

#### Reach

A median of 1.7% [an interquartile range (IQR): 1.3, 2.4%] residents in recruited villages was screened with stroke history within 50 eligible villages. At the village level, the proportion of people recruited in the trial accounted for a median of 70.1% (IQR: 58.3, 87.%) among all stroke survivors screened in the villages. The recruited participants were similar to those screened with self-reported stroke history in the region, except stroke survivors who reported bedridden were generally not recruited. The participants could represent a general group of rural community-dwelling stroke survivors with a median of 5.3 (IQR: 2.3, 9.8) years of stroke history since the first event, stable health conditions, and basic communication abilities (Table 2). The findings from the in-depth interviews revealed that knowledgeable village doctors who reviewed existing health records and performed door-to-door screening enabled the reach of the program to the targeted population in a timely fashion. People employed outside of the villages or having families living outside may be left out of the program (Table 4).

#### Adoption of the SINEMA Program Among Village Doctors

Fifty eligible villages out of 109 villages within five townships were formally invited and recruited in the study. These villages represented typical middle-to-large-sized rural villages of Northern China with a median of 2,422.5 residents per village (IQR: 1,772, 3,600) (Supplementary Table 2). All village doctors (*n* = 25, mean age: 46 ± years old, 12% females, Table 3) from the intervention arm adopted the SINEMA intervention. A few village doctors expressed that they had seldomly used mobile apps before the program and experienced a learning curve. Some village doctors did not use all modules of the SINEMA App due to lacking needs and motivations.

*“I seldomly used the performance module and checked these statistics. I only check whether I missed any follow-up visits. I completed my tasks while evaluating my work is other people's tasks.” —Village doctor, 6-month interview*

Twenty village doctors (83.3%) considered the training sessions as the most valuable and essential component. They believed that knowledge and skills gained through training sessions and previous experience in delivering similar services mitigated the learning curves of program adoption and enabled them to deliver the follow-up visit to the participants. The follow-up visits module and the training modules of the SINEMA App received 14 and 13 votes, ranked as the second and third most essential components by village doctors. Financial incentives were not ranked highly as essential intervention components, as some village doctors mentioned that the financial incentives did not impact much on their decisions of program adoption or the amount was not high enough to be a driver; rather, the perceived benefits to the residents that village doctors learned from the communication with county and township physicians, as well as the opportunities of receiving training and guidance from experts, were major enablers that determined their adoption.

*“The payment didn't influence me much. It was not the case that if I got more money, I could work better…. Even if you stop paying me, it will not influence me much. Similar to Basic Public Health Services, the project brought benefits to our residents. After participating in this project, I could better manage my patients; I met them face-to-face monthly. If someone comes to check my work, I do not need to lie to him or her, as I have done this work as required. There is a benefit.”—Village doctor, 9-month interview*

#### Implementation and Fidelity of Follow-Up Visits

Twenty-five village doctors in the intervention arm performed an average of 291.5 (SD: 29.5) follow-up visits over 12 months. Among 637 participants, 564 (88.5%) received no <12 follow-up visits as full dose per protocol. The participants who received the full dose were more likely to be those without family caregivers and had multiple chronic disease conditions (Supplementary Table 3). Although the quantity of the follow-up visits was high, the quality of follow-up visits varied. Follow-up visits were delivered mainly at the village clinics or homes of patients. Some village doctors scheduled all follow-up visits on certain days of a month; others performed the follow-up visits as an add-on service once participants came to clinics. Based on descriptions of village doctors of the key steps of follow-up visits, we found that many village doctors adjusted the procedure by skipping some steps.

*“I opened the app, measured the blood pressure, and asked questions based on the app. If needed, I asked all questions (on the app), or I selected key questions to ask. …For example, if the patient has a stable situation, or I know him or her quite well, I may skip the question about hospitalization and only emphasize medicine use. For the side effect of medicines, it does not need to be asked each time.”—Village doctor, 6-month interview*

Factors that influenced the quantity and the quality of follow-up visits are summarized in Table 4. Internal enablers included perceived responsibilities from providers, good preexisting patient-doctor relationship, and strong willingness from the participants. The SINEMA App and the quality control from the township physicians promoted the intervention fidelity. Village doctors mentioned that the designed SINEMA App played a supportive role in standardizing follow-up visits and assisting the information management. However, the required internet access may also bring some barriers when there is no stable internet access. Village doctors and township physicians also stated that preexisting heavy workload and lacking compliance of patients might limit the quantity and the quality of follow-up visits, but the top-down support and quality control may encourage high fidelity.

*“He (the township physician) is playing supervision and encouraging role. For example, if he found that there are certain patients that left without follow-up visits, he will remind me to finish at my earliest.” -Village doctor, 12-month interview*

#### Implementation and Acceptance of Voice Messages

About half of the participants answered the voice messages during the program implementation on a given day among those who agreed to receive voice messages. The answering rate was maintained over the 12-month implementation period (Supplementary Figure 1). The implementation of voice messages was influenced by phone use patterns and characteristics of the participants. Both quantitative and qualitative data indicated that the participants who had their phones without sharing with family members picked up more voice messages (Supplementary Table 3). Most participants mentioned that they considered voice messages as good reminders and a reliable source of getting information and favored the simple content, repeated leading sentences, and local dialect. However, some participants who experienced cognitive declines could only recall the leading sentences. In contrast, a few participants stated that they dropped out of the voice-message component halfway through as the contents were too simple for them.

*“It (the voice messages) says about taking medicines and doing exercise. I cannot remember other details…*. *Nevertheless, it is useful as it reminds me in the morning, and it shows care about me.” –Participant, 12-month interview*

#### Maintenance

During the interview, most of the village doctors and the participants expressed their willingness to continue the program. Some village doctors suggested expanding the participants to individuals with other chronic conditions, but others also expressed concerns about the workloads if the program expanded to a larger population group. Village doctors perceived that impacts of intervention could be maintained as participants have established good habits in visiting village clinics and taking medicines. Some village doctors also expressed a spillover effect of the program to other existing services and programs.

*“During these months, they have developed a habit. Participants have kept a good relationship with me, and I expect they will continue visiting me as often…. I also used this program approach for the Basic Public Health Services. I planned for the follow-up visits regularly so that I don't need to be rushed or to lie to people who check my work.”- Village doctor, 9-month interview*

The county and township managers also expressed that the maintenance of the program may be influenced by the scope of targeted participants, workloads of village doctors, and the financial mechanisms to cover the cost of the program delivery. Integrating the program with the existing programs and information systems was also mentioned as critical factors for future maintenance and scaling-up.

*“This program should be integrated with other activities in both urban and rural settings. Such as the program should be integrated with the basic public health services so that the program could get more policy support.” -Township physician, 12-month interview*

### Effectiveness and Perceived Mechanism of Change

The trial results on the effectiveness of the SINEMA intervention have been detailed elsewhere (12). In brief, the intervention achieved a significant reduction in systolic blood pressure (between-arm difference: −2.8 mmHg, 95% CI: −4.8, −0.9; *p* = 0.005), and improved medication adherence, physical activities and quality of life as secondary outcomes and reduced stroke recurrence, hospitalization, and deaths as exploratory outcomes. The impact pathways of the intervention were further revealed through in-depth interviews.

#### The Influence on Confidence and Practice of Village Doctors

Village doctors acknowledged that they prescribed medicines mainly by following the previous prescriptions before the intervention. Training and support improved their awareness of clinical guidelines and encouraged them to provide more guidance and suggestions during visits of patients. Some village doctors also mentioned that they considered adherence and long-term benefits of participants while prescribing medicines. The survey at 12 months post-baseline also indicated increased perceived confidence in prescribing evidence-based medicines and supporting patients (Table 3).

*“Through this project and the training session, I changed my mind by considering not only the medicine price but also the effect. For patients who had poor adherence, I suggested them to change to prolonged antihypertensive medicines. Although the cost is a little bit higher, the effect in controlling blood pressure was largely improved.”—Village doctor, 9-month interview*

#### Interactive Impact of Voice Messages and Follow-Up Visits on Self-Management and Doctor-Patient Relationships of Patients

An interactive function between voice messages and follow-up visits has been identified from interviews. We found that the face-to-face communication between providers and participants about the content of voice messages facilitated the general adoption and acceptance of the voice message component; meanwhile, the daily voice messages reinforced adherence of patients to the suggestions of the doctors delivered during the follow-up visit component. Village doctors stated that voice messages supplemented their role to provide extra assistant for the participants to improve their treatment adherence. Some participants mentioned that they discussed the contents of voice messages with village doctors, which reinforced them to pick up voice messages and translate health education information to self-management activities, as they received consistent information from the village doctors and voice messages.

*“The voice messages improved their awareness. We (village doctors) cannot observe and remind patients every day, but we can only remind patients when we have a face-to-face appointment. The voice messages add to that to remind patients so that they consider how they should take medicines and do exercise as the doctors told them; then, they establish a good habit.”—Village doctor, 9-month interview*

The frequent face-to-face visit and daily voice messages also help village doctors build or maintain a good relationship with the participants. About 88% of the village doctors acknowledged that their patients relied more on them due to the intervention, and the project improved their authorities in the village (Table 3). The patients also mentioned that they felt more care from the village doctors through voice messages and follow-up visits.

*“Through the follow-up visits, patients trust me more. (I conducted) follow-up visits once a month, without asking them to pay, and, if they don't come, I will call them to remind them. There were several patients who sought care from other providers if they were sick (before the intervention). But I followed up with them and they said they were willing to my suggestions because they think my suggestions were helpful. Now, our relationship is not bad. I say “hi” to them if I meet them on the street.”*—*Village doctor, 12-month interview*

*“The doctor measured my blood pressure frequently. He also reminded me to pick up the call or visit him if I forgot. I could feel that he cares about me.”—Participant, 9-month interview*

#### The Influence of Personal and Interpersonal Characteristics on Program Effectiveness

The influence of follow-up visits and voice messages varied by person. Personal factors, such as personal education background, cognitive functions, and self-efficacy, may influence their acceptance of information delivered, the impact on behavior changes as intermediate outcomes, and the long-term effects on health outcomes.

*“Almost all of them could understand the content, but some people, such as those who have received education or with strong willingness to improve their health, could absorb more information from voice messages; for others, they may just listen to them but not take anything from them.”—Village doctor, 3-month interview*

*“This disease cannot be fully treated. I am becoming older, and I don't think I could get better. Even if I don't have a disease, my health condition will get worse anyhow.”- Participant, 12-month interview*

Village doctors also mentioned that family caregivers are another channel that they exchange information with if family caregivers could become the extra support to encourage participants to adhere to the treatment and self-management activities.

*“Some stroke patients had impaired brain function. It is hard to communicate with them…For these patients, I told their family members about their medicine prescriptions to remind them. But there is also a case that the patients' wife spends the whole day playing mahjong and seldomly take care of the patient. For this type of patients, their family are not helpful at all.” -Village doctors, 12-month interview*

#### The Impact of Other Healthcare Services or Programs

Some participants mentioned that they also sought healthcare services from other healthcare providers. Inconsistent information gained from different sources brought obstacles for participants to adhere to the suggestions provided by village doctors during the follow-up visits, thus may limit the role of the village doctors.

*“I take medicines from several places (providers). I could pay less (out of pocket) from other places if I know the person.”—Participant, 12-month interview*

*“About half of participants visited county hospitals and got their medicines prescribed there because they could get reimbursement if they have special non-communicable disease insurance. For them, I don't interrupt their medicine use*—*I only encourage them to continue taking medicine.”—Village doctor, 9-month interview*

Besides, some other existing services or programs had some overlap with the SINEMA intervention, as the participants with hypertension and diabetes could have been targeted by these programs already. Although not all the participants involved in the trial could access other services, it may attenuate the observed between-arm differences of the intervention.

*“There are other health education and follow-up visits services as part of the Essential Public Health Services. We provided village-wide health education sessions and advocated basic public health services, including blood pressure management. Our residents (including stroke patients) may improve their health literacy and be aware of the benefit of taking medicines and keep a good diet (during the study period due to these programs).” —Village doctors from the control arm, 12-month interview*

## Discussion

Based on the RE-AIM and MRC Process Evaluation frameworks, this study gives new evidence concerning implementation outcomes and the factors relevant to the implementation and effectiveness of the SINEMA program. The SINEMA intervention successfully reached a representative group of community-dwelling stroke survivors and brought significant benefits to health and wellbeing of participants. Although some of the village doctors made adaptations to the program delivery approach, all of them adopted the intervention and delivered with high fidelity. Some potential impact pathways were identified, such as empowerment among village doctors in clinical decision-making and the interactive impact on stroke survivors *via* both human-delivered services and technology-enabled components. The contextual factors, including personal and interpersonal characteristics and healthcare system and environment, also interacted with intervention components and provided some further explanations about program effectiveness.

For complex interventions, implementation and process evaluation is very important to provide additional information on how the different intervention components were implemented and interacted with the context. We innovatively derived a framework from both the RE-AIM and the MRC process evaluation frameworks to provide a more comprehensive view of program implementation and effectiveness. Following the recent suggestion about the use of RE-AIM framework (17), we used mixed methods to report both the findings on each dimension of RE-AIM and illustrated the facilitators and barriers that may influence these dimensions. The MRC framework provided another lens through which we are able to understand the context and potential impact pathways for the effects of the SINEMA program. The study findings provide learnings for policymakers, health practitioners, and researchers regarding the future adaptation, optimization, and implementation of the SINEMA program.

Our findings demonstrate that future adaptation of the SINEMA intervention needs to consider the coherence and relationship of its intervention components. Many of the SINEMA intervention components, such as capacity building, task shifting, home-based follow-up visits, and technology-enabled tools, have been investigated previously (9, 28–32). However, the impact of these components was not always positive and additive in the real-world setting (15). Indeed, our study demonstrates that the intervention components were not independent of one another but rather interacted and were synergistic with each other. The components of training, financial compensation, and top-down support from township physicians became the facilitators for adopting and implementing the follow-up visit component. The follow-up visits and voice message components interactively influenced behaviors of providers and patients, and improved doctor-patient relationships. This finding re-emphasized the core concept of the Chronic Care Model, that is, the importance of building a provider-patient alliance with productive information exchange (33, 34). This suggests that future implementation of community-based services should address the barriers of program adoption at levels of both providers and patients by combining effective strategies into a streamlined program to flat the adoption curve and to overcome implementation challenges.

Our study also emphasized the unique role of information and communication technologies in supporting program delivery and program evaluation. The use of information and communication tools generated data to support intensive monitoring of program implementation. Future studies could further investigate the best approach of using digital solution-generated data for providing real-time feedback about program implementation. The digital health system also supported intervention delivery. In line with previous studies (35), we also identified barriers to digital health adoption among individuals who shared devices or had low-technology literacy. Interestingly, our study also showed a reinforcement loop between the technology-enabled component and the human-delivered component that were not considered in many previous studies (36–38). Such findings indicated some spillover effect of digital health solutions beyond service delivery if the technology-enabled component could be embedded into the healthcare system. The results added further evidence on mHealth-enabled interventions on chronic disease management, whose importance was amplified due to the COVID-19 pandemic (39).

Findings from our study also illustrated the complex impact of stakeholders and the context on program implementation and effectiveness. Variability of program implementation across villages was attributed to attitude, capacities, and practice of village doctors. In line with previous studies that identified barriers to normalizing new interventions when a competing program exists (15, 40, 41), we also noticed the challenge to sustain SINEMA intervention when primary healthcare providers rated their workloads as high with limited capabilities and incentives in stretching to other new tasks. However, our study illustrated the benefits of the SINEMA intervention to existing services in reducing the learning curve and the spillover effect of the SINEMA intervention on improving the quality of existing preventive services. This finding highlighted the importance of considering the synergies and integration across programs when introducing and implementing a new program.

The impact of the intervention was different among subgroups. For example, the quantitative subgroup analysis showed that the impact of the SINEMA intervention was consistently positive except for males and those who were <65 years (12), while the qualitative data added further information by showing that different self-efficacy, doctor-patient relationships, and preference in service utilization may explain the variation in health outcomes. Males and younger participants are more likely to take some casual work outside of villages and had more chance to interact with other healthcare providers beyond the intervention scope, such as private health clinics or pharmacies. As the qualitative findings suggested, services provided from these facilities may disrupt the uptake or the intervention impact pathway when conflicting information was delivered to the participants. These findings provide more explanations about program effectiveness and offer suggestions for future program optimization to deliver more personalized information and involve other available private services in the program.

Our study has several strengths. We developed an evaluation framework that combined the RE-AIM and MRC frameworks and used data collected at four time points to comprehensively understand the implementation of the SINEMA intervention. Data extracted from the digital health system provided additional real-time monitoring on program uptake and implementation beyond the traditional observational approach. In addition, we combined the findings from quantitative and qualitative data to demonstrate how the program was implemented and to identify the facilitators and barriers that may influence the program implementation and effectiveness.

There were also some limitations. First, the definitions and measurement of RE-AIM indicators were defined based on the best estimation of available data. For example, we measured the program reach by analyzing the representativeness of our participants among screened stroke survivors in the study settings rather than more broad scope due to the limited access of data. The measurement of program maintenance is also limited to willingness of the participants to maintain the program, which may overestimate the long-term program maintenance in actual practice. Second, the trial was conducted in 50 villages of one county in rural Northern China. Villages and participants recruited in the trial shared many similarities, limiting the observed diversity and the external validity of the study findings. Future studies could explore the adaptation and implementation of the program when the program is disseminated to diverse settings and populations. Third, as a common limitation of complex intervention, we could not fully explain the impact mechanism or distinguish separate effects from different components. However, we illustrated some potential impact pathways that were not explained by quantitative intermediate outcomes and provided some unexpected pathways or consequence that influenced the impact. Such findings are important for considering the scale-up and adaptation of the program to other contexts and settings.

## Conclusion

The SINEMA intervention reached a representative stroke patient group in rural China, adopted by village doctors and implemented with relatively high fidelity. The program benefited both providers and patients, but the impact was diverse by characteristics of individual, interpersonal relationships, and other services in the setting. There is a need to explore the adaption of the SINEMA model in other settings and for other chronic diseases.

## Data Availability Statement

The raw data supporting the conclusions of this article will be made available by the authors, without undue reservation.

## Ethics Statement

The studies involving human participants were reviewed and approved by Duke University Ethical Review Board, Duke Kunshan University Ethical Review Board, Beijing Tiantan Hospital Ethical Review Board. The patients/participants provided their written informed consent to participate in this study.

## Author Contributions

EG drafted the manuscript. EG, WG, QL, and LY contributed to the conceptualization, design of the study, and data collection. JB and LY contributed to acquiring study funding. EG, LS, and JT performed data analysis. EG, LS, HX, QL, and LY were involved in the data interpretation. QL, HX, JB, JM, TJ, BO, and LY contributed to the revision of the manuscript. All authors read and approved the final manuscript.

## Funding

The study is funded by the United Kingdom Medical Research Council, Economic and Social Research Council, Department for International Development, and Welcome Trust (Grant No. MR/N015967/1). EG is supported by the Melbourne Graduate Scholarship. EG and BO were also supported by the National Health Medical Research Council (Grant 1170937). The publication of this article is supported by Shandong Center for Disease Control and Prevention, School of Population Medicine and Public Health, Peking Union Medical College.

## Conflict of Interest

The authors declare that the research was conducted in the absence of any commercial or financial relationships that could be construed as a potential conflict of interest.

## Publisher's Note

All claims expressed in this article are solely those of the authors and do not necessarily represent those of their affiliated organizations, or those of the publisher, the editors and the reviewers. Any product that may be evaluated in this article, or claim that may be made by its manufacturer, is not guaranteed or endorsed by the publisher.
