# Supplementary material for: The Implementation of a Primary Care-Based Integrated Mobile Health Intervention for Stroke Management in Rural China: Mixed-Methods Process Evaluation
Source: Front Public Health. 2021 Nov 17;9:774907. doi: 10.3389/fpubh.2021.774907 (PMC8635640; doi:10.3389/fpubh.2021.774907)

**Supplement Table 1. The number of in-depth interviews completed and fully analyzed at each wave of the process evaluation**

| **Stakeholders** | **3-months**  **(n/n)** | **6-months**  **(n/n)** | **9-months**  **(n/n)** | **12-months**  **(n/n)** |
| --- | --- | --- | --- | --- |
| **Participants (stroke patients)** | 5/16 | 4/8 | 4/9 | 6/18 |
| **Village doctors in the intervention arm** | 2/10 | 4/7 | 4/6 | 6/10 |
| **Village doctors in the control arm** | 0/0 | 0/0 | 0/0 | 1/2 |
| **Township physicians** | 2/5 | 1/2 | 0/0 | 2/3 |
| **County coordinators** | 1/1 | 0/0 | 0/0 | 1/1 |

n/n: number of interviews fully analysis /number of interviews completed

**Supplement Table 2 Characteristics of villages that involved in the screening, trial and implemented the SINEMA intervention**

| **Villages-level characteristics** | **Participated in the screening**  **(n=60)** | **Participated in the SINEMA trial**  **(n=50)** | **Implemented the SINEMA Intervention**  **(n=25)** |
| --- | --- | --- | --- |
| **Number of residents in each village, median (Q1, Q3) *** | 2117.5  (1664, 3283) | 2422.5  (1772, 3600) | 2289  (1787, 3,331) |
| **Distance between village clinic to the county hospital, km** |  | 14.5 (4.8) | 14.4 (4.9) |
| **Number of stroke patients screened, median (Q1, Q3)** | 34 (25, 45.5) | 37 (26, 50) | 33 (25, 43) |
| ≤20 | 5 (8.3%) | 0 (0.0%) | 0 |
| 21-30 | 19 (31.7%) | 16 (32.0%) | 10 (40.0%) |
| 31-40 | 16 (26.7%) | 14 (28.0%) | 7 (28.0%) |
| >40 | 20 (33.3%) | 20 (40.0%) | 8 (32.0%) |
| **Number of village doctors** |  |  |  |
| 1 | 6 (10.0%) | 5 (10%) | 5 (20%) |
| 2-3 | 30 (50.0%) | 26 (52.0%) | 14 (56.0%) |
| 4-5 | 18 (30.0%) | 14 (28.0%) | 5 (20.0%) |
| >5 | 6 (10.0%) | 5 (10.0%) | 1 (4.0%) |

**Supplement Table 3. Baseline characteristics of participants who achieved a high implementation fidelity**

|  | **Participants allocated into intervention arm**  **(n=637)** | **Participants received more than half of dispatched voice messages**  **(n=362)** | **Participants completed 12 or more follow-up visits**  **(n=564)** |
| --- | --- | --- | --- |
| **Socio-Demographic characteristics** | | | |
| **Age, years** | 66.2 (8.2) | 65.3 (8.0) | 66.1 (7.9) |
| **Sex, % female** | 272 (42.7%) | 150 (41.4%) | 238 (42.2%) |
| **Education** |  |  |  |
| No schooling | 264 (41.4%) | 144 (39.8%) | 232 (41.1%) |
| Some schooling or primary school only | 182 (28.6%) | 102 (28.2%) | 160 (28.4%) |
| Above primary school | 191 (30.0%) | 116 (32.0%) | 172 (30.5%) |
| **Marital Status** |  |  |  |
| Married | 526 (82.6%) | 305 (84.3%) | 471 (83.5%) |
| Widowed, divorced or not married | 111 (17.4%) | 57 (15.8%) | 93 (16.5%) |
| **Having a family caregiver** | 410 (64.3%) | 224 (61.9%) | 205 (36.4%)** |
| **Access to mobile phone** |  |  |  |
| **Phone ownership** |  |  |  |
| No phone (may have a shared phone) | 164 (25.7%) | 79 (21.8%) | 146 (25.9%) |
| Basic phone | 435 (68.3%) | 266 (73.5%)** | 385 (68.3%) |
| Smartphone | 38 (6.0%) | 17 (4.7%) | 33 (5.9%) |
| **Sharing a phone with family members** | 187 (39.5%) | 99 (35.0)** | 161 (38.5%) |
| **Health conditions at baseline** | | | |
| **Stroke type** |  |  |  |
| Ischemic | 555 (87.1%) | 307 (84.8%) | 485 (86.0%) |
| Hemorrhage | 80 (12.6%) | 54 (14.9%) | 77 (13.7%) |
| Not specified | 2 (0.3%) | 1 (0.3%) | 2 (0.4%) |
| **Self-report diseases** |  |  |  |
| Hypertension | 461 (72.4%) | 264 (72.9%) | 410 (72.7%) |
| Dyslipidemia | 248 (38.9%) | 146 (40.3%) | 217 (38.5%) |
| Diabetes | 113 (17.7%) | 71 (19.6%) | 102 (18.1%) |
| Heart Diseases | 70 (11.0%) | 43 (11.9%) | 57 (10.1%)** |
| **Health Outcomes at baseline** | | | |
| **Systolic blood pressure, mmHg** | 146.02 (20.9) | 145.7 (21.0) | 146.4 (20.5) |
| **Diastolic blood pressure, mmHg** | 77.98 (11.6) | 78.3 (11.9) | 78.0 (11.6) |
| **Health-related quality of life score, range 0-1** § | 0.80 (0.2) | 0.80 (0.2) | 0.81 (0.2) |
| **Achieving a level of health enhancing physical activity** | 99 (15.5%) | 59 (16.3%) | 91 (16.1%) |
| **Medication use** |  |  |  |
| Antiplatelet | 432 (67.8%) | 253 (70.0%) | 384 (68.1%) |
| Statin | 158 (24.8%) | 91 (25.1%) | 135 (23.9%) |
| Anti-hypertensive medicines | 522 (81.9%) | 298 (82.3%) | 467 (82.8%) |
| **Adherence to medications** † |  |  |  |
| Antiplatelet | 275 (63.7%) | 154 (60.9%) | 245 (63.8%) |
| Statin | 106 (67.1%) | 60 (65.9%) | 95 (70.4%) |
| Anti-hypertensive medicines | 329 (63.0%) | 185 (62.1%) | 293 (62.7%) |
| **Moderate to severe disability** ¶¶ | 179 (28.1%) | 96 (26.5%) | 150 (26.6%) |
| **Stroke hospitalization in the past year** | 124 (19.5%) | 62 (17.1%) | 104 (18.5%) |

******** *Significant differences were observed between the group with all participants who allocated into the intervention arm (p<0.05)*

**Supplement Figure 1. Response rate of participants who received the voice message over the intervention implementation period.**


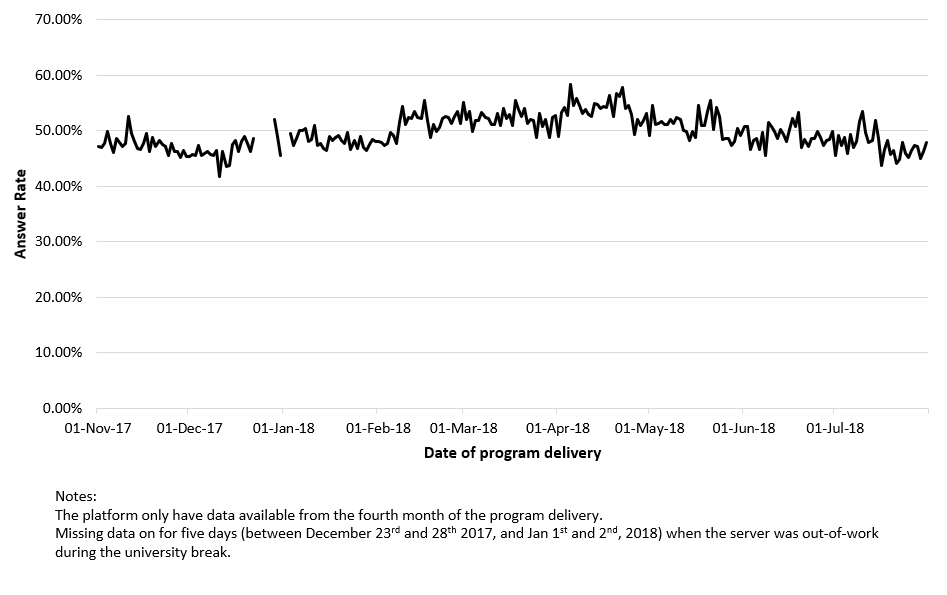

Supplement: Supplementary file 1 [file Data_Sheet_1.docx]
